# Effects of Reserve Capacity on Seismic Response of Concentrically Braced Frames by Considering Brace Failure

**DOI:** 10.3390/ma15134377

**Published:** 2022-06-21

**Authors:** Zengyang Zhao, Wenyuan Zhang, Yukun Ding, Hongwei Li

**Affiliations:** 1 Key Lab of Structures Dynamic Behavior and Control of the Ministry of Education, Harbin Institute of Technology, Harbin 150090, China; hitzhaozy@foxmail.com (Z.Z.); dingyukun@sohu.com (Y.D.); lihongwei.hit@foxmail.com (H.L.); 2 Key Lab of Smart Prevention and Mitigation of Civil Engineering Disasters of the Ministry of Industry and Information Technology, Harbin Institute of Technology, Harbin 150090, China

**Keywords:** concentrically braced frames, low-ductility systems, reserve capacity, brace failure, nonlinear time-history analysis

## Abstract

In order to study the influence of brace failure on the seismic response of concentrically braced frames and the improvement of the residual structure’s resistance to collapse due to reserve capacity, a series of concentrically braced frame prototypes with different story numbers is designed. A matrix of six finite-element concentrically braced-frame (CBF) models is established, which is varied by the number of stories and the level of reserve capacities. Accuracy of the numerical model is verified by comparing the responses of the shaking-table test of the concentrically braced frames, under 10 different working conditions. Then, a nonlinear time-history analysis, considering brace failure in one specified story, is carried out. The results show that the story-drift angle of the failure story as well as its adjacent stories increases greatly in the ideal pinned model. The above phenomenon is particularly serious, when the failure occurs at the top or bottom of the structure. With the reserve capacity brought by column continuity, and the semi-rigid rotation capacity of the beam-to-column and column-to-base connections are taken into consideration, the increase in story-drift angle caused by the brace failure is effectively reduced. However, the inherent reserve capacity has little influence on the dynamic characteristics of concentrically braced frames in the elastic stage.

## 1. Introduction

A steel concentrically braced frame (CBF) is one of the efficient and commonly used lateral-load-resisting systems, with its work lines essentially intersecting at points [1]. The steel brace offers high lateral stiffness for drift control and reduces the material consumption of the structure [2]. The seismic response of such frames is, therefore, dominated by the asymmetric axial resistance of the bracing members, which is due to the influence of the following physical phenomena: yielding in tension, buckling in compression, post-buckling deterioration of compressive load capacity, deterioration of axial stiffness, and low-cycle fatigue fractures at the plastic-hinge regions [3]. The other parts, such as the beams and columns, are generally designed to remain elastic [4].

If the CBF is regarded as ideal pinned, in strict accordance with the design assumption, the structure will become a mechanism and collapse immediately after its brace fails. In fact, despite a brace failure, a large number of steel frames (including gravity frames and braced frames) were found to have not collapsed during the 1985 Mexico earthquake [5], the 1994 Northridge earthquake [6], and the 1995 Hyogo-ken Nanbu earthquake [7]. Preliminary studies suggests that these structures may avoid collapse, not because of the integrity of the primary lateral-force-resisting system [8] (LFRS, i.e., brace system in CBFs), but because the residual structure possesses reserve capacity [9,10].

A variety of research on the reserve capacity in CBF structures is underway, and the potential sources can be basically summarized into two aspects: structure members and their connections. Brace-gusset plates can provide beam-to-column connections with a substantial-moment capacity of up to 90–100% of the plastic-moment capacity of the beam (*M_p,beam_*) [11,12]. William A. analyzed the mechanism, where the gusset plate exerted an extrusion effect on the connected beams and columns [13]. Christopher D. pointed out that the braced-gusset plate not only increased the flexural strength and stiffness of the structure, but also improved the collapse resistance of the structure [14]. Similarly, floor slabs not only contribute greatly to the initial stiffness of the structure [15] but also can provide asymmetric beam-to-column moment capacity until the crushing of concrete by 0.04 rad-story drift [16,17]. Even the theoretically pinned connections, such as beam–column connections employing a shear tab, double angle, and end-plate details, which are common in a gravity system, can develop roughly 15–20% of *M_p,beam_* on their own [14,18,19]. At the same time, the existence of connected angle steel and end plates also enhances the energy-dissipation capacity of the panel zone [20]. Moreover, since the anchor bolts are usually arranged outside the column cross-section, to carry the gravity loads plus the overturning from the seismic loads, a semi-rigid column base with moment capacity is formed [21,22]. It should also be noted that columns are vertical continuous members across floors in practical structures. Supplemented by the moment capacity, column continuity can also provide a reliable reserve capacity, when braces or their connections experience brittle failure in an isolated story, making the residual structure works like a moment steel frame within the failure story [8,23].

Generally, a reserve system is more flexible than a primary LFRS, hence, unlike the redundancy provided by extra LFRS elements, the reserve capacity activates when the primary LFRS sustains significant damage [10]. As a philosophical concept, reserve systems have the potential to increase safety, reduce construction cost, and encourage innovation in LFRS design.

In general, various researches on the reserve capacity of CBFs mainly focus on quantifying the reserve capacity from different potential sources. By contrast, research on these sources that comes together to affect the seismic response of the whole structure is quite minimal. Thus, the primary objective of this paper is to determine the influence of reserve capacity on the dynamic characteristics and seismic response of CBFs. Four sources of reserve capacity are specifically considered: (1) gravity connections; (2) braced connections; (3) base fixity; and (4) column continuity. Another objective is to explore the influence of brace failure in an isolated story on CBFs with and without added reserve capacity.

At present, nonlinear time-history analysis based on the finite-element model is an effective means to study the seismic performance of structures [24,25]. This paper will fulfill these objectives through nonlinear time-history analysis. Firstly, 4- and 10-story CBF buildings, which are loaded by Chinese seismic-design code [26], are designed in accordance with Chinese steel-structure-design code [27] and seismic provision of AISC [28]. Secondly, traditional pinned models are abstracted from the CBF prototypes, and, on this basis, the connection-modeling method is changed to consider different degrees of reserve capacity. After that, the brace is set to fail and stop working at different failure times. By comparing the dynamic characteristics and seismic responses of the residual structures of different models, the effect of reserve capacity on the seismic performance of a CBF structure is analyzed.

## 2. Development and Validation of CBF Models

### 2.1. Prototype Design

**Figure 1:**
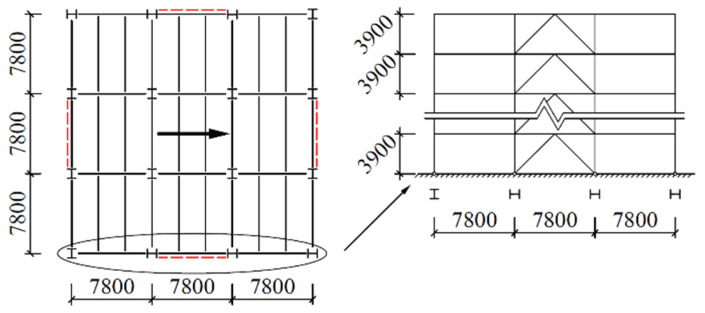
Plan view and elevation of CBF prototypes (unit: mm).

Concentrically braced frames are designed for 4- and 10-story building configurations. Each design has a square floor plan with dimensions of 23.4 m by 23.4 m (three bays at 7.8 m by three bays at 7.8 m), along with 3.9 m story heights. The prototype building includes two interior braced frames in each direction, for a total of four braced bays per level. See Figure 1 for a plan view and the bracing elevations. The red dotted lines in Figure 1 represent the position of the braces.

Members were sized using a live load of 3.5 kN/m^2^ and a dead load of 5.0 kN/m^2^. The base shear of each model is computed under a design basic-ground-motion acceleration of 0.2 g, plus other parameters such as first-design earthquake classification, site class II, and building class C, based on the Chinese seismic-design code [26], are considered. The considered seismic action direction is shown by the bold arrow in Figure 1.

Table 1 and Table 2 list the member sizes for each design. Column 2 lists the braced frame columns, designed to carry both gravity loads and overturning forces. Column 3 lists the braced-frame girders. Column 4 lists the brace sizes. All braces are designed as a welded I-section, field welded to the gusset plates, with the web vertical to the ground. The width-to-thickness-ratio grade of a brace plate is BS3 [27], which is equivalent to the design requirement of a brace in an OCBF structure [28]. Braces designed in accordance with these provisions are expected to provide limited-inelastic-deformation capacity and are vulnerable to low-cycle-fatigue failure under seismic loads. Column 5 lists the gravity-frame columns, designed to carry gravity loads only. Column 6 lists the gravity frame beams. Specified steel grades are Q355B (*f*_y_ = 355 MPa) for the beams and columns and Q235B (*f*_y_ = 235 MPa) for the braces.

### 2.2. Finite-Element Model

Finite-element simulations through the commercial program ABAQUS [29] are conducted to establish analytical models for the CBF structures identified in Table 1 and Table 2. Taking advantage of the symmetry of the prototype building’s floor plan, the numerical simulations are performed in one direction with a braced bay attached to two “leaning” gravity columns and a unidirectional excitation is considered. The Timoshenko beam element, with shear deformation (B32), was used to simulate all the beam, column, and brace members. The classical metal-plasticity model is used with an elasticity modulus of *E* = 2.06 × 10^5^ MPa and Poisson’s ratio of 0.30. Considering the material nonlinearity, bilinear-kinematic-hardening behavior is adopted. The tangent modulus after yield is taken as 0.2% *E*. A half-sine-wave sweep was induced into each brace, with a peak initial imperfection of 1/1000 times the brace length for buckling prediction. Considering the structural nonlinearity (P-delta effect [30]), the geometric nonlinear switch (Nlgeom) is turned on during the whole time of the nonlinear time-history analysis.

**Figure 2:**
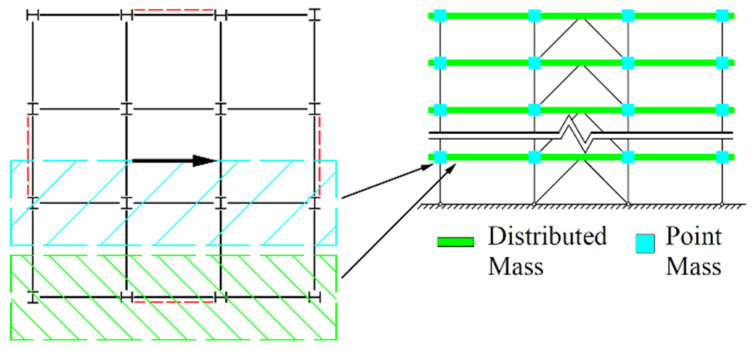
Allocation of floor mass.

Though all floor members are ignored during the modelling process to reduce computational costs, their effects should be accurately captured, which could basically be divided into two aspects: restricting members’ out-of-plane torsion and providing inertia force under lateral loads. For the former, the out-of-plane displacement of the beam and column elements is constrained. For the latter, the non-structural distributed and point “Mass” elements are arranged, as shown in Figure 2, to achieve the equivalence of the representative value of gravity load. “Non-structural” refers to this kind of element that produces inertial force in the direction of seismic load, without any additional vertical load. The mass on the subordinate area of the brace span is transformed into the representative value of the gravity load and evenly distributed to the corresponding beams. The gravity-span counterpart is applied to the corresponding column of the brace span, in the form of point mass.

**Figure 3:**
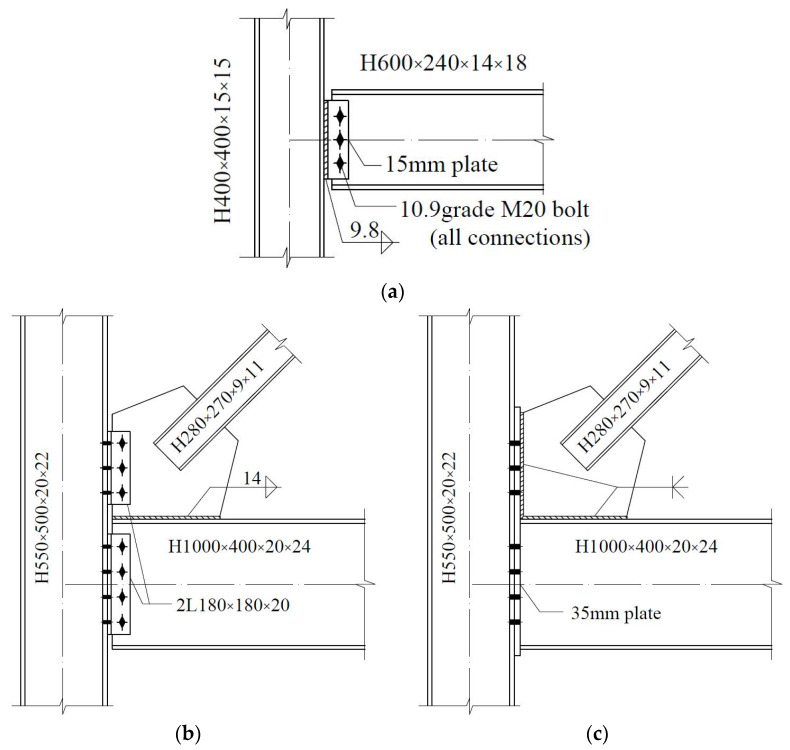
Connection details: (**a**) shear-tab connation; (**b**) double-angle connection; (**c**) end-plate connection (unit: mm).

**Figure 4:**
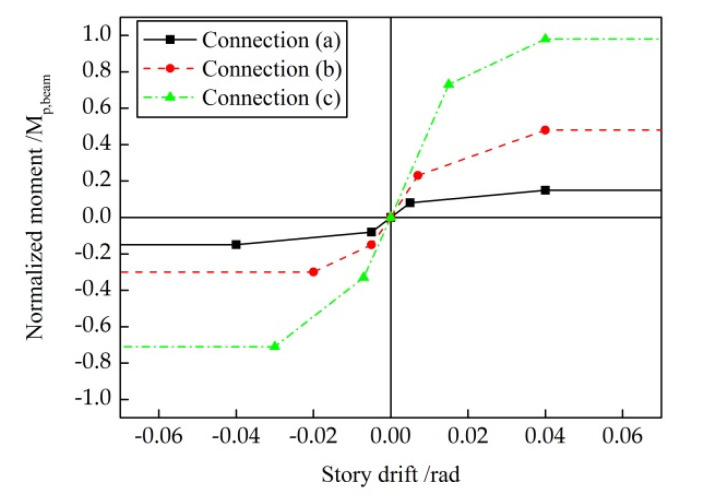
Connection models in S1 and S2.

Three models are established for each CBF prototype. The baseline model, which is named P1, represents a case where all of the members are connected by pinned connections that would traditionally be assumed in the analysis and design of CBFs. A large amount of research shows that these pinned connections do have more or less inherent lateral resistance to form the reserve capacity, employing different details. Therefore, to explore the effects of reserve capacity on seismic response, three typical connections with the details shown in Figure 3 are selected, and two semi-rigid models, S1 and S2, containing varies levels of reserve capacity, are established. A summary of the connection type is provided in Table 3. Nonlinear connector sections are used to capture the semi-rigidity of the connections in both the gravity and braced frames. Available test and simulation results (Stoakes, C.D. [11] and Liu, J. [17]) are used as a benchmark to calculate the moment and rotation relations of the connections. The calculated moment relative to their respective beam-plastic moments vs. the rotation curves for each connection are shown in Figure 4. The bottom of model P1 constrains the displacement in two directions in the plane and relaxes the rotation constraint in the plane; the bottom ends of models S1 and S2 constrain not only the displacement in two directions in the plane but also the rotation in the plane.

### 2.3. CBF Model Validation

The finite-element CBF model was validated using a shake-table test conducted previously [31]; this reference experimental program was summarized here for clarity.

**Figure 5:**
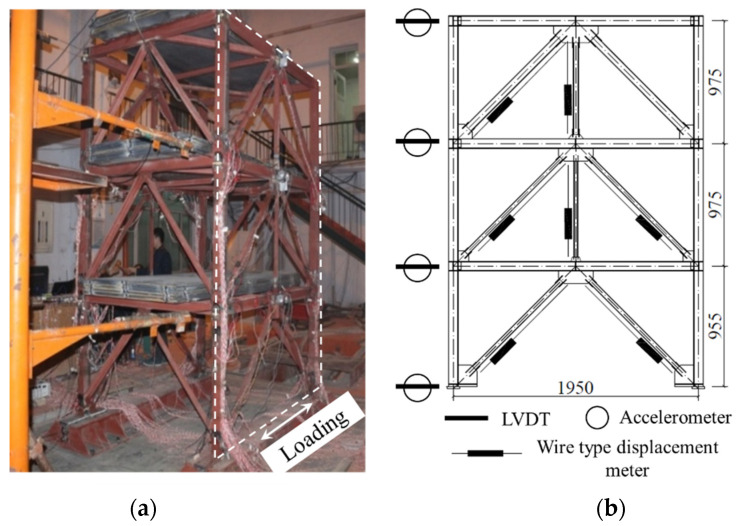
Shake-table test of concentrically braced frame (unit: mm). (**a**) Test setup; (**b**) Elevation.

The test specimen was based on a three-story CBF with a design basic-ground-motion acceleration of 0.2 g, and the test setup was shown in Figure 5. The scaling rules for the shake-table test were summarized in Table 4. The frame comprised welded H-section beams (*d* = 78, *b_f_* = 65, *t_w_* = 3.75, *t_f_* = 3.75 mm), welded H-section columns (*d* = 78, *b_f_* = 78, *t_w_* = 3.75, *t_f_* = 3.75 mm), and welded H-section braces (see Table 5 for the detailed dimensions). The beam webs were welded to the column flanges through the shear tabs, to achieve pinned beam-to-column connections. The column base plates were connected to heavy base beams through high-strength bolts, which in turn were pinned and connected to the shake table. For each material comprising the specimen, Table 6 lists the material-test results.

**Figure 6:**
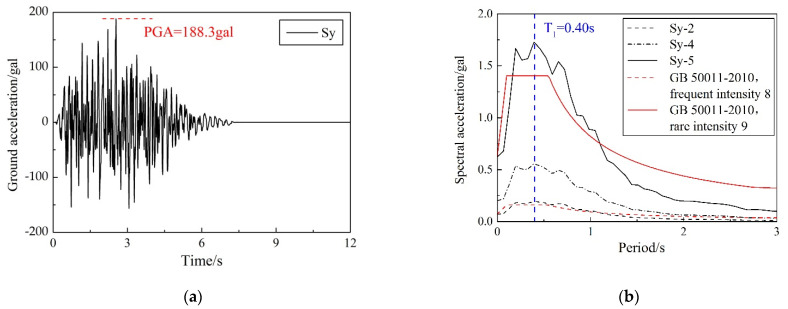
Ground motion Sy: (**a**) acceleration history; (**b**) acceleration-response spectrum.

When selecting a reasonable ground-motion record, the spectral characteristics of ground motion were mainly considered. By scaling the ground-motion records, the acceleration-response spectrum of the selected record was equal to or slightly higher than the corresponding code-design-response spectrum. The Sy motion was introduced in the direction shown in Figure 5a. Sy, characterized by a peak acceleration of 188.3 gal and strong velocity pulses, was a synthetic motion created to match the GB 50011-2010 site class II 8-degree frequent-intensity earthquake spectrum. Figure 6a showed the acceleration history of the motion, whereas Figure 6b showed the acceleration-response spectrum. Shake-table tests were conducted by introducing the Sy motion several times, with the target amplification level increasing from 181.3 gal, 538.0 gal, 1668.0 gal, and 2218.4 gal, to, finally, 2351.9 gal, which represents the earthquake action from 8-degree frequent intensity and 8-degree basic intensity to 9-degree rare intensity and continuous, strong earthquake action. The frequent, basic, and rare intensity were the seismic intensity with the exceedance probability of 63%, 10%, and 2~3% in the 50-year-design reference period, respectively. Detailed loading protocol is summarized in Table 7.

**Figure 7:**
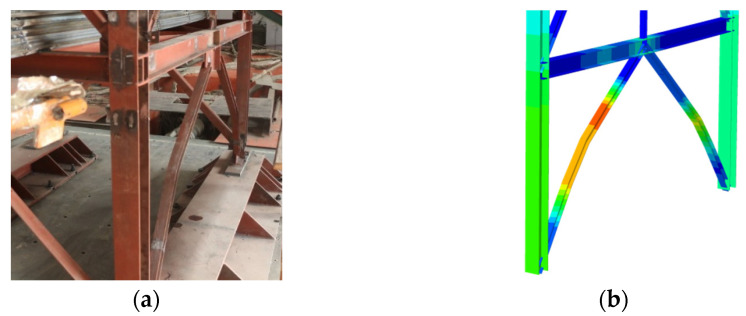
Comparison of specimen-failure phenomena: (**a**) test; (**b**) simulate.

Using the method introduced in the previous section, a finite-element CBF model was established according to the member size of the shake-table test specimen, and a nonlinear time-history analysis under the same loading protocol was carried out. Based on the last continuous, strong ground motion (Sy-10) of the shake-table test, the failure mode of the numerical model was compared with the test specimen. At that time, the 1st- and 2nd-story braces had great out-of-plane residual deformation, while the whole structure did not collapse, which reflected the residual seismic-bearing capacity. It was obvious from Figure 7 that the failure mode of the finite-element model was very close to the test specimen, which was due to the serious out-of-plane deformation of the bottom braces.

**Figure 8:**
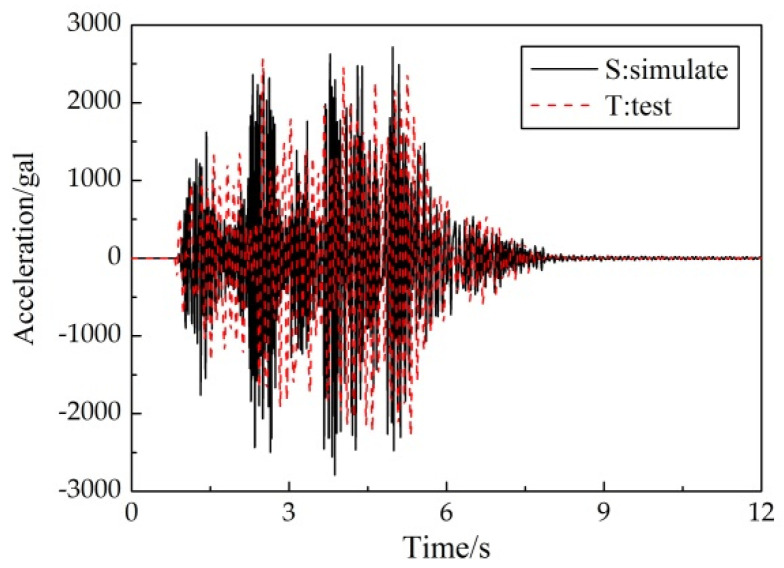
Comparison of roof-acceleration response under Sy-2 condition.

**Figure 9:**
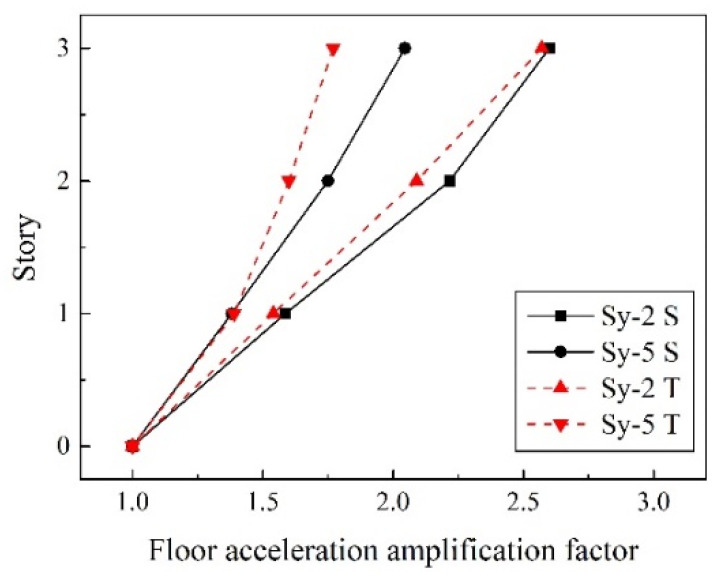
Comparison of FAA under different conditions.

Figure 8 illustrated the roof-acceleration response under 8-degree frequent-intensity ground motion (Sy-2) of the test specimen and the numerical model. Figure 9 plots the distribution of the floor-acceleration amplification (FAA) under 8-degree frequent-intensity (Sy-2) and 9-degree rare-intensity (Sy-5) ground motions. As illustrated, the roof-acceleration response of the finite-element models correlated well with the experimental results. The FAA of each story was also well represented. In case of an8-degree frequent earthquake, the FAA increased with the story number. Under the condition of a 9-degree rare earthquake, the overall level of FAA decreased, which showed that with the enhancement of earthquake intensity, plastic deformation occurred and reduced the acceleration response of each story.

**Figure 10:**
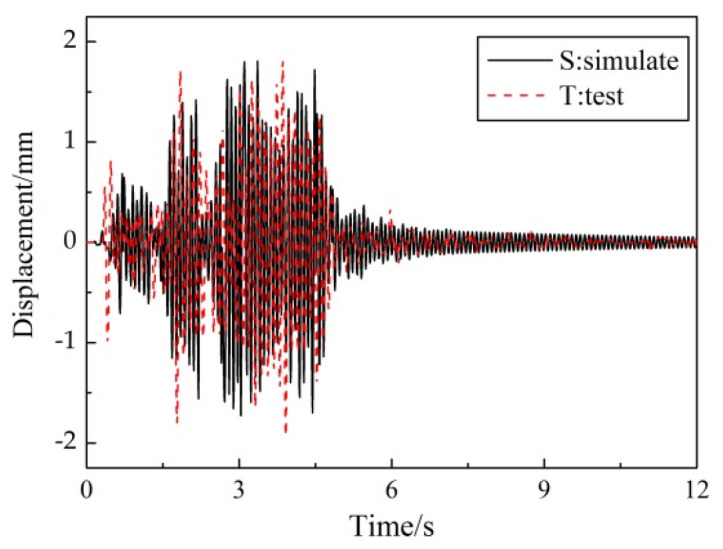
Comparison of roof-displacement response under Sy-2 condition.

**Figure 11:**
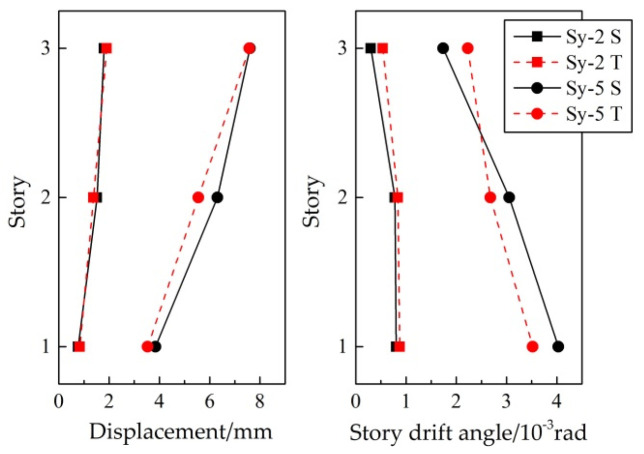
Comparison of structure overall response under different conditions.

Figure 10 illustrated the roof-displacement response under the 8-degree frequent-intensity ground motion (Sy-2) of the test specimen and the numerical model. Figure 11 compared the envelope diagram of displacement and story drift under 8-degree frequent-intensity (Sy-2) and 9-degree rare-intensity (Sy-5) ground motions. The figure showed that the simulation traced the experimental response fairly accurately. Similarly, the maximum value of the story drift appeared in the bottom story, which was easy to form a weak story.

In a brief summary of finite-element-model validation, the simulated results had a good agreement with the shake-table test results, which indicated that the proposed models could capture the major behaviors of the CBF structures under different earthquake levels and provided us with confidence in using the models for the subsequent analysis.

## 3. Nonlinear Time-History-Analysis Strategy

**Figure 12:**
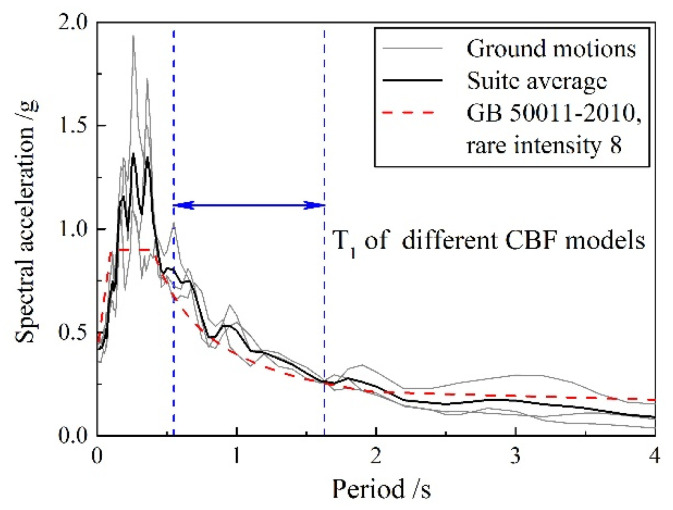
Ground-motion-suite response spectra.

To examine the change in behavior under dynamic loading, the 4- and 10-story CBF prototype buildings are subjected to a suite of three earthquake ground-motion time histories, corresponding to 2–3% probability of exceedance in 50 years for a first-design earthquake classification, with site class II outlined by GB 50011-2010. All the records are selected from the ground-motion database of the Pacific Earthquake Engineering Research (PEER) Center. Response spectra for the three ground motions and the suite average are plotted in Figure 12.

Owing to the brittle mechanisms inherent in the R = 3 CBF system, the typical limit state encountered in this prototype is low-cycle-fatigue failure of the braces and their connections followed by drift-induced degradation. Pre-analysis of numerical model constructed incorporating fracture that can initiate according to a calibrated rain-flow-count rule shows that the cumulative-fatigue-damage value of the brace after a single time-history analysis is about 0.1 to 0.2, which is far away from the low-cycle-fatigue limit (the cumulative-fatigue-damage value reaches 1 [32]). This is due to the fact that it is difficult to consider the unexpected machining and constructing defects. These adverse effects will not only result in the fatigue-damage value of the brace being seriously underestimated but also lead to more random brace-failure locations. Since the fracture cannot be accurately anticipated by the computer simulation, engineering judgment had to be exercised.

In order to explore the influence of brace failure at different positions on the seismic response of the residual structure, three brace-failure times (*t_bf_* = 5 s, 10 s and 15 s) are defined, which correspond to before, during, and after the occurrence of the peak acceleration of the selected ground-motion suite, representing that the failed brace has different low-cycle-fatigue lives. According to the brace-failure time, the ground motions are divided into three parts, and the nonlinear time-history analysis incorporating brace failure is achieved as follows: ① introduce the amplitude-modulated ground motions at the column base and record the interaction force between the proposed failed brace as well as the surrounding components during the previous analysis step of the brace-failure time; ② remove all of the failed brace elements at the brace-failure time defined above, and the interaction force recorded before gradually decays to 0 within the next second, representing the process of the failed brace from the low-cycle-fatigue-crack initiation to complete the fracture; and ③ sequentially, the nonlinear time-history analysis of the remainder of the structure under subsequent ground motions is continued.

## 4. Results Analysis

### 4.1. Comparison of Dynamic Characteristics

The first three-order period of each model is shown in Table 8. The fundamental periods of the 4-story buildings are 0.570 s, 0.561 s, and 0.557 s, respectively, for models P1, S1, and S2; in the 10-story buildings, the fundamental periods for the three cases are 1.630 s, 1.592 s, and 1.568 s, respectively. The observation indicating that the influence of the rotation capacity of beam-to-column and column-to-base on the initial stiffness is negligible, which reinforces the conclusion that the reserve capacity does not come into play until the primary LFRS sustains significant damage.

**Figure 13:**
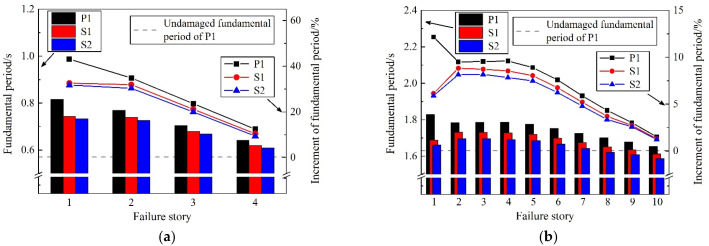
Fundamental period of residual structure. (**a**) 4-story. (**b**) 10-story.

Although the initial system is quite stiff, with a small fundamental period, this undamaged fundamental period of CBF does not heavily govern its response in a seismic event. Figure 13 shows the fundamental period of the remainder after one brace fails, and the increment compared to its intact counterpart. The bar chart in the lower part shows the fundamental period of the residual models after the brace fails in different stories, while the line chart in the upper part shows the increment of different fundamental periods compared with the undamaged ones. As shown, failure of even just one brace of the LFRS decreases the stiffness of the building and increases its fundamental period significantly. For the four-story CBF systems investigated, the semi-rigid frames outperformed the pinned counterparts regardless of connection type. This difference is most pronounced for the condition when the first-story braces fail, in which the fundamental period increment for P1 is 43.13%, compared with 32.57% and 31.74% for S1 and S2. This result is directly related to the inherent reserve capacity brought by the semi-rigid flexural stiffness of beam-to-column and column-to-base connections. With the increase in the number of story failures, the increment of the fundamental period compared with the undamaged structures decreases gradually, indicating that the upper brace has a relatively weak impact on the overall stiffness of the structure. A similar trend was seen for 10-story buildings.

**Figure 14:**
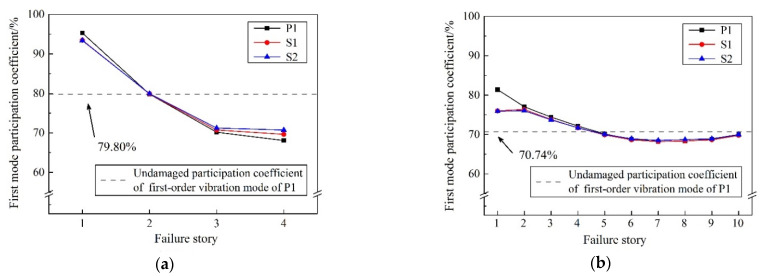
First-mode participation coefficient of residual structure. (**a**) 4-story; (**b**) 10-story.

Figure 14 shows the participation coefficients of the first-order vibration mode (*Γ*_1_) of the remainder after one brace fails. In four-story CBFs, *Γ*_1_ jumps to 95.28%, 93.46% and 93.37%, respectively, for models P1, S1, and S2, after the first story has failed. Therefore, the first story is critical and governs the behavior of the structure under subsequent ground motions. It is worth mentioning that when the brace fails in the upper stories, that is the third and fourth stories in a 4-story CBF and the sixth to tenth stories in a 10-story CBF, *Γ*_1_ drops below an undamaged value: 79.80% and 70.74% for 4-story and 10-story CBFs, respectively. Accordingly, the proportion of higher-order modes increases, and the upper stories of the remaining structure may suffer additional seismic damage, as will be seen in more detail below.

Overall, the result from the modal analysis shows that inherent reserve capacity aids the remainder of a CBF structure in partly maintaining its original stiffness after the brace failure. On this foundation, further nonlinear time-history analysis is conducted to explore nuances that cannot be discerned in modal analysis.

### 4.2. Comparison of Seismic Response in Failure Story

**Figure 15:**
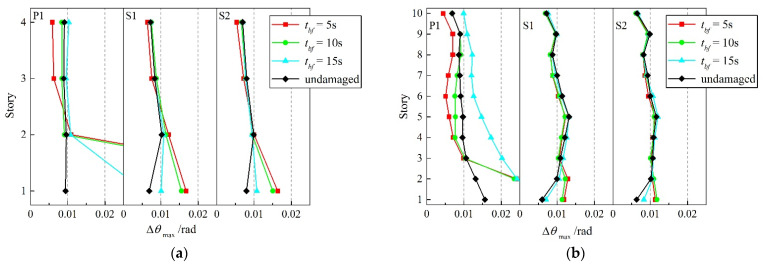
Δ*θ*_max_ of residual structure after first-story brace fails. (**a**) 4-story; (**b**) 10-story.

A set of nonlinear time-history analyses are conducted per the CBF models established. The results from these analyses are aggregated into mean values for each prototype configuration, to quantify the reserve capacity’s effect on the remainder of the structure after the brace failure. Since the gap of the dynamic characteristics in the condition when the first story brace fails stands out from the previous analysis, a comparison of the maximum story-drift angle (Δ*θ*_max_) for each modified model is shown in Figure 15.

Under a severe earthquake, seismic codes in many countries tend to adopt the method of limiting the story drift rather than the structure strength, to prevent the overall collapse. A 0.02 rad threshold is defined as the collapse point for multistory and high-rise steel structures in the Code for Seismic Design of Buildings (GB 50011-2010) [20]. After the first brace fails, the failure story enters a long-link eccentrically braced frame (EBF) mechanism. The ductility of this mechanism is limited to 0.01 rad in the affected story, based on the preliminary results from a braced-frame test at the National Center for Research in Earthquake Engineering in Taiwan [33], which roughly represents another drift-related limitation of the CBF structure.

As the figure shows, the brace failure in the first story at the beginning 10 s of the ground motions eventually leads the residual model to the non-convergence of the time-integration scheme, which clearly represents the actual phenomenon of dynamic collapse. With the delay of brace-failure time, though the residual model turns back to numerical stability, the Δ*θ*_max_ of the first story in the residual model still reaches over 0.03 rad, obviously exceeding the limit value of the elasto-plastic story-drift angle specified in GB 50011-2010. The reserve capacity gained when modeling the column-to-base connection as fixed plays an important role in the behavior of the residual structure, after the primary LFRS has failed in the first story. The severe numerical instability following when the first story fails in P1 is corrected, and the Δ*θ*_max_ of the first story is controlled at 0.007 rad and 0.008 rad for 10-story S1 and S2, respectively, after the first story fails at 15 s, which not only prevents the collapse of the structure but also forms a reliable EBF mechanism. For the mechanism that develops, the reserve capacity gained from enhancing the beam-to-column connections is small. This observation is consistent with previous modal analysis, that the first mode of participation mass accounts for 95.28% and 81.39% for 4- and 10-story residual structures, respectively, and column bending dominates the behavior after a brace fails in the first story.

**Figure 16:**
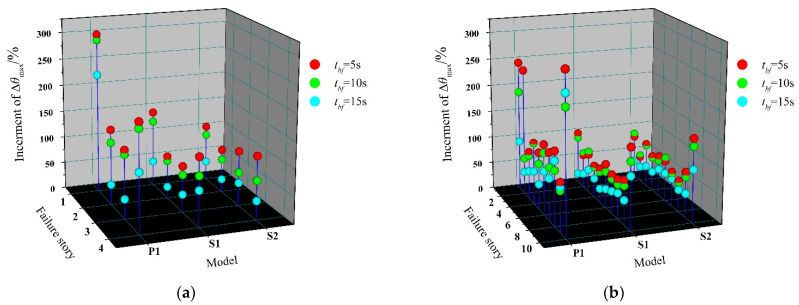
Increment of Δ*θ*_max_ in failure story: (**a**) 4-story; (**b**) 10-story.

Considering the unexpected defects in practical engineering, the brace of any story may take the risk of fracture during a strong earthquake. Hence, the increment of Δ*θ*_max_ in the failure story, relative to the undamaged structure under the failure of different story braces, is further explored (Figure 16). When the first-story brace fails at a very early time, the increment of the first-story Δ*θ*_max_ in P1 reaches over 250%. In this case, the benefit of the reserve capacity is realized as it limits the increment of the first-story Δ*θ*_max_ in S2 to 105.02% and 79.21% for 4- and 10-story residual structures, respectively. What’s more, this increment falls to 34.76% and 30.71%, respectively, when *t_bf_* = 15 s, effectively preventing a soft-story mechanism, which leads to collapse in the baseline case after the initial failure of the LFRS. Increment of the failure story Δ*θ*_max_, under the condition where the brace fails in the middle part of the structure, is relatively average, distributed mostly in the range of 30–80%.

Note that the increment of the top-story Δ*θ*_max_ after the brace fails shows an abnormal uplift. As the figure reveals, the increment of the top-story Δ*θ*_max_ in P1 reaches 200.99% and 295.34% for 4- and 10-story, respectively, after the brace fails in the top story when *t_bf_* = 5 s. S2 still has the increment of the top story Δ*θ*_max_ up to 118.59% and 151.05% for 4- and 10-story residual structures, respectively, in the same case. The abnormality of the top story is not a novel observation, with a formation that is similar to the whiplash effect, to a certain extent. Due to the usual small section of members in the top story, there will be a huge lateral-stiffness gap between the top story and the lower parts, after the brace fails. Thus, in the process of the structure swinging forth and back, it is easy to generate a large velocity in this very story, resulting in a large inter-story drift. This observation can be corroborated by the phenomenon of the relatively larger proportion of higher-order modes, compared to the undamaged structure in the previous modal analysis. What is a relief is that the structure is unlikely to suffer much damage, since the seismic force exerted on the top story is very small, and the possibility of failure of a brace in a top story is very low.

On the whole, with the delay of brace-failure time, the increment of the maximum story-drift angle decreases gradually. After one brace fails, the inherent reserve capacity created by the enhanced beam-to-column and column-to-base connection keeps the stiffness of the failure story in the residual structure at a certain level, so as to avoid the formation of a soft-story mechanism and improve the seismic performance.

### 4.3. Comparison of Seismic Response of Residual Structures

**Figure 17:**
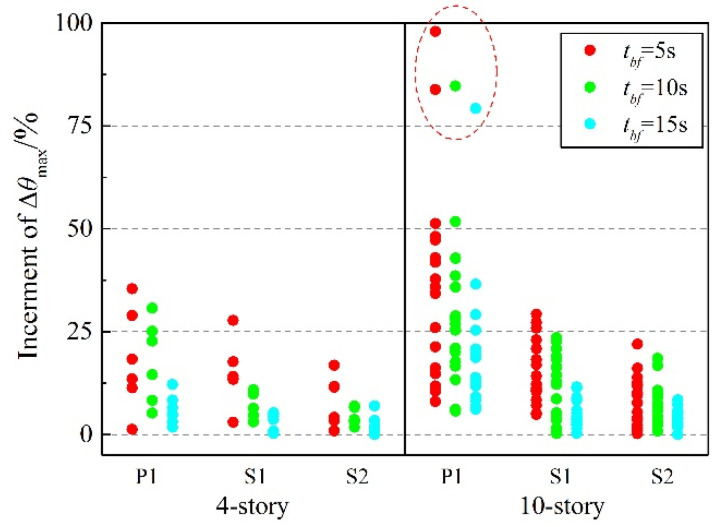
Increment of Δ*θ*_max_ in adjacent story of failure story.

Based on the observed behavior of the engineering judgment, brace failure not only seriously weakens the stiffness of the failure story but also causes the stiffness of adjacent stories to decrease, which is not conducive to the integrity of the structure. Figure 17 shows the increment of the Δ*θ*_max_ in the adjacent (upper or lower) stories of failure story to be relative to the undamaged structure under the failure of different story braces. For the 10-story P1, after the failure of the first-story brace under the condition where *t_bf_* = 5 s, 10 s, and 15 s, the Δ*θ*_max_ of the second story is 0.0241 rad, 0.0235 rad, and 0.0243 rad, and the corresponding increment of Δ*θ*_max_ is 83.85%, 84.68% and 79.21%; after the failure of the second-story brace under the condition where *t_bf_* = 5 s, the Δ*θ*_max_ of first story is 0.0260 rad, and the corresponding increment of Δ*θ*_max_ is 97.95%. The Δ*θ*_max_ of these four prominent data points (circled by dotted lines in Figure 17) exceeds the limit value of the elasto-plastic story-drift angle specified in GB 50011-2010, which means that a soft-story mechanism caused by brace failure faded away from the failure story into the adjacent undamaged stories.

Excluding the above four points (circled by red dotted line in Figure 17), the increment of the Δ*θ*_max_ in the adjacent stories of the failure story still has relatively large discretion, reaching over 50%. With the enhanced beam-to-column connection and column-to-base connection taken into consideration, the increment of the Δ*θ*_max_ in the adjacent stories becomes more concentrated, with a maximum of 29.22% and 21.91% for S1 and S2, respectively. On the premise of the vertical continuity of columns, the stiffness and strength of the enhanced connections can aid in the development of a frame-action mechanism following a limit state within the LFRS, such as brace buckling or a brace-connection fracture. Like a dual system, a frame action mechanism can distribute an inelastic response over multiple stories, reduce concentrations of story drift, and limit the further spread of damage.

The drift concentration factor (*DCF*), given in Equation (1), is often used to evaluate the lateral displacement mode of structures [34], which describes the ratio of the maximum Δ*θ*_max_ to the maximum roof drift, *u*_r,max_/*H*, where *u*_r,max_ = maximum roof displacement and *H* = structure height. This ratio is unity, when the frame moves over linearly with height. Otherwise, it is greater than unity [35]. The closer the *DCF* is to 1.0, the more the inter-story-drift angle of each story of the structure tends to be consistent, the better the control effect of the overall lateral deformation mode of the structure is, and the more the seismic capacity of each member can be brought into full play [36].

*DCF* = max{Δ*θ*_max_}/(*u*_r,max_/*H*) (1)

**Figure 18:**
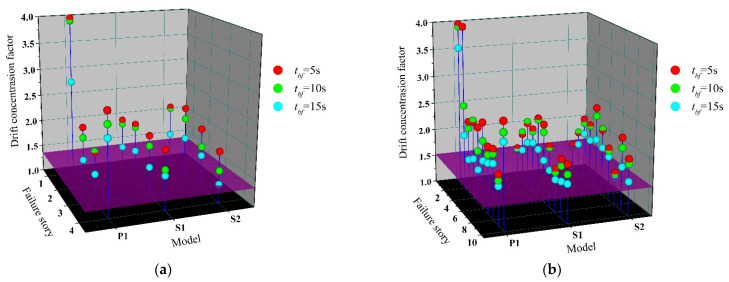
Drift concentration factor of residual structure. (**a**) 4-story; (**b**) 10-story.

Figure 18 shows the *DCF* of the residual CBF structure after one brace fails. Similar to the previous modal analysis, the influence of enhanced two connections on *DCF* is not obvious in the undamaged structure, which has a *DCF* of 1.347 and 1.522 for 4-story and 10-story prototype buildings, respectively, and is represented by a plane parallel to the ground. After the brace fails, the *DCF* increases in varying degrees. In accordance with expectations, after the failure of the first-story brace of P1, the increment of story drift caused by the damage is concentrated near the failure story, and the *DCF* reaches to a considerable value, representing the overall collapse of the residual structure.

Thanks to the better overall performance brought by enhanced beam-to-column and column-to-base connections, the *DCF* after brace failure of S1 and S2 is basically concentrated in the range of 1.5–2. Especially after the failure of first-story and top-story braces, the *DCF* value of the residual structure decreases significantly compared with P1. The semi-rigid connection makes the flexural stiffness of the vertical continuous columns play to a greater extent, which resists the tendency for concentration of deformation in one story, improves the capacity of the system to redistribute the demand over the building height, and becomes a reliable source of reserve capacity after the brace failure.

## 5. Discussion

In the present study, an elasto-plastic time-history analysis of low-ductility concentrically braced frames, considering brace failure, is carried out. Analysis results demonstrate that reserve capacity can effectively improve the collapse resistance of residual structures after the brace failure. In the reports of major earthquake damage during the past decades, a large number of concentrically braced-frame steel-structure buildings have not collapsed, even though the cracking failures at beam-to-column connections as well as brace-to-beam and column connections have been found. The analysis results of this paper confirm the existence of this phenomenon and give an explanation from the mechanism level.

Even for concentrically braced-frame structures with low ductility (braces and their connections do not meet the seismic-detailing requirements), the whole system will not collapse at one touch after some braces have failed because of large earthquakes, and the residual structure still has a certain anti-collapse capacity. In other words, if the inherent reserve capacity of the structure can be properly utilized after brace failure, the economy of this kind of structure will be greatly improved, especially in areas of low and moderate seismicity. This also promotes the update and progress of the Minimum Design Loads for Buildings and Other Structures (ASCE/SEI 7-16) [37]. The R = 3 provision for steel structures in low- and moderate-seismic regions, which allows for seismic force reduction without ductile detailing, implicitly relies on reserve capacity for collapse prevention.

In this paper, four sources of reserve capacity are considered: (1) gravity connections; (2) braced connections; (3) base fixity; and (4) column continuity. However, there are many other sources for the reserve capacity of the concentrically braced-frame structure, including but not limited to the improvement of the connection performance of the composite floor and the brace re-engagement mechanism. This requires us to propose a new and more comprehensive connection skeleton model to consider this part of the reserve capacity. If the failure and collapse mechanism of the low-ductility concentrically braced-frame structure can be deeply studied, the applicable scope (intensity, height, etc.) under different construction details can be built, and a design method that can reasonably consider the reserve capacity can be put forward; thus, the low-ductility concentrically braced-frame structure is bound to have a wide application prospect in the industrial and civil buildings of low- and moderate-seismic regions.

## 6. Conclusions

(1) Enhanced beam-to-column and column-to-base connections have little effect on the fundamental period (T_1_) of undamaged CBF structures, which is slightly reduced by 1.58–3.80% compared with the ideal pinned counterpart. However, the inherent reserve capacity is helpful for the remaining structure to maintain the original dynamic characteristics. The structural damage caused by brace failure will increase the T_1_ of residual structures, especially when the first-story brace fails. Reserve capacity could maintain this increment of T_1_ within 31.74% and 8.83% for 4-story and 10-story residual structures, respectively, after brace failure. Moreover, the failure of the first-story brace will greatly increase the participation coefficients of the first-order vibration mode (*Γ*_1_), which is 98.28% and 81.39% for 4-story and 10-story structures, respectively, and dominates the subsequent seismic response of the residual structures.
(2) Although the semi-rigid connection has little influence on the dynamic characteristics of the undamaged CBF structures, it can bring a significant seismic reserve capacity after the brace failure in the elasto-plastic stage, which can substantially reduce the increment of maximum story-drift angle (Δ*θ*_max_) in residual structures. After the first-story brace fails at a very early time in the ideal pinner CBF structure, a soft-story mechanism is formed, and there is a high probability of overall collapse. While for the case of enhanced beam-to-column and column-to-base connections, the Δ*θ*_max_ of the failure story is maintained within 0.02 rad, and the corresponding increment compared with the undamaged ones is kept below 80.47% and 64.65% for 4-story and 10-story prototypes, respectively, which is conducive to avoid the overall collapse of the residual structures.
(3) The inherent reserve capacity originates from the semi-rigid connections and vertical continuous columns of the CBF structure that can also limit the increment of adjacent story drift caused by brace failure within 25%, prevent concentration of deformation, promote redistribution of the stiffness and displacement demand over the building height, and improve the overall seismic performance of the residual structure. For the case of semi-rigid connections, the drift-concentration factor (*DCF*) of the residual structure after brace failure is basically concentrated in the range of 1.5–2, which, from another point of view, proves that the overall deformation performance of the CBF structure, when considering the reserve capacity, is much better.
(4) With the increase in the rotational stiffness of the beam-to-column and column-to-base connections as well as the delay of the brace-failure time, the influence of brace failure on the seismic response of the residual structures after brace failure is gradually weakened, and the risk of overall collapse of the residual structure is reduced. The exemption from the seismic-detailing requirement in an *R* = 3 CBF structure makes it impossible and unnecessary for designers to delay or even avoid the failure of the brace under a severe earthquake, by improving its low-cycle-fatigue life. However, the inherent reserve capacity could be a useful tool for preventing collapse of the CBF structures after the brace failure in moderate seismic regions.

## Figures and Tables

**Table 1:** Design-member sizes of four-story prototype (unit: mm).

Story	Braced Frame			Gravity Frame	
	Column	Girder	Brace	Column	Beam
4	H400 × 400 × 15 × 15	H950 × 360 × 18 × 22	H245 × 210 × 7 × 9	H400 × 400 × 15 × 15	H600 × 240 × 14 × 18
3	H400 × 400 × 15 × 15	H1000 × 400 × 20 × 24	H260 × 230 × 8 × 10	H400 × 400 × 15 × 15	H600 × 240 × 14 × 18
2	H400 × 400 × 15 × 15	H1000 × 400 × 20 × 24	H275 × 255 × 8.5 × 10	H400 × 400 × 15 × 15	H600 × 240 × 14 × 18
1	H400 × 400 × 15 × 15	H1050 × 400 × 20 × 24	H280 × 270 × 9 × 11	H400 × 400 × 15 × 15	H600 × 240 × 14 × 18

**Table 2:** Design-member sizes of 10-story prototype (unit: mm).

Story	Braced Frame			Gravity Frame	
	Column	Girder	Brace	Column	Beam
10	H400 × 400 × 15 × 15	H950 × 360 × 18 × 22	H245 × 210 × 7 × 9	H400 × 400 × 15 × 15	H600 × 240 × 14 × 18
9	H400 × 400 × 15 × 15	H950 × 360 × 18 × 22	H260 × 220 × 7 × 9	H400 × 400 × 15 × 15	H600 × 240 × 14 × 18
8	H550 × 500 × 20 × 22	H1000 × 400 × 20 × 24	H260 × 230 × 8 × 10	H400 × 400 × 15 × 15	H600 × 240 × 14 × 18
7	H550 × 500 × 20 × 22	H1000 × 400 × 20 × 24	H260 × 230 × 8 × 10	H400 × 400 × 15 × 15	H600 × 240 × 14 × 18
6	H550 × 500 × 20 × 22	H1000 × 400 × 20 × 24	H270 × 245 × 8 × 10	H400 × 400 × 15 × 15	H600 × 240 × 14 × 18
5	H550 × 500 × 20 × 22	H1000 × 400 × 20 × 24	H270 × 245 × 8 × 10	H400 × 400 × 15 × 15	H600 × 240 × 14 × 18
4	H600 × 550 × 22 × 24	H1000 × 400 × 20 × 24	H275 × 255 × 8.5 × 10	H400 × 400 × 15 × 15	H600 × 240 × 14 × 18
3	H600 × 550 × 22 × 24	H1050 × 400 × 20 × 24	H280 × 270 × 9 × 11	H400 × 400 × 15 × 15	H600 × 240 × 14 × 18
2	H700 × 650 × 24 × 26	H1050 × 400 × 20 × 24	H280 × 270 × 9 × 11	H400 × 400 × 15 × 15	H600 × 240 × 14 × 18
1	H700 × 650 × 24 × 26	H1050 × 400 × 20 × 24	H280 × 270 × 9 × 11	H400 × 400 × 15 × 15	H600 × 240 × 14 × 18

**Table 3:** Connection-type summary of CBF models.

	Model	P1	S1	S2
Connection				
Gravity frame	beam-to-column	pin	a	a
	column-to-base	pin	rigid	rigid
Braced frame	brace-to-frame	pin	pin	pin
	beam-to-column	pin	b	c
	column-to-base	pin	rigid	rigid

**Table 4:** Scaling rules of the CBF shake-table test.

Physical Property	Parameter	Scaling Factor
Geometry	Length	1/4
Material	Stress	1/1
	Elastic modulus	1/1
	Poisson’s ratio	1/1
	Mass	1/43.04
Load	Story shear	1/16
Dynamic	Time	1/3.28
	Velocity	1/1.22
	Acceleration	2.69/1

**Table 5:** Parameters of braces and gusset plates connected at both ends.

Story	Brace	Width-to-Thickness Ratio (Slenderness Ratio)	Corner Gusset Plate						Mid-Span Gusset Plate						
			a	b	c	e	t	Sketch	a	b	c	d	e	t	Sketch
3	H62 × 62 × 3.75 × 3.75	5.8 (111)	114.5	56.6	82	0 (0t)	8	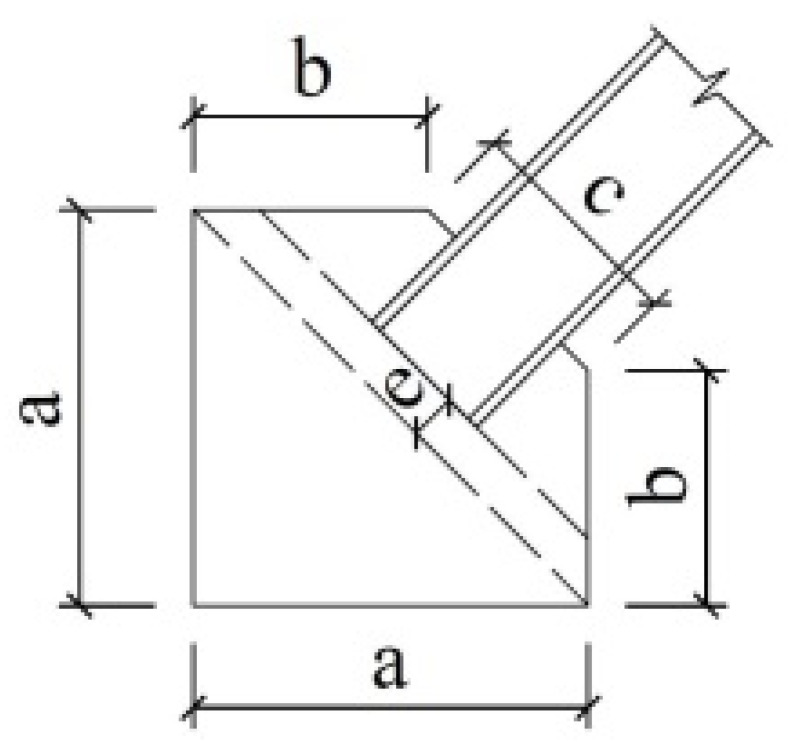	268.3	37.2	82	152.4	16 (2t)	8	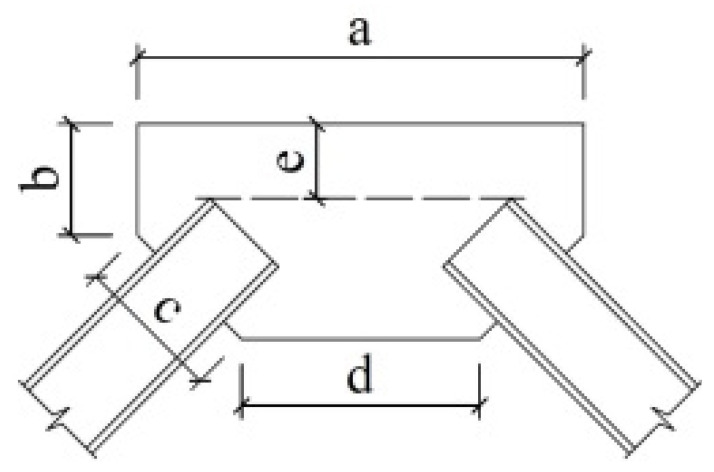
2	H35 × 35 × 3 × 3	5.3 (120)	101.0	60.0	58	12 (2t)	6		308.0	74.0	55	226.0	60 (10t)	6	
1	H38 × 38 × 3 × 3	7.8 (70.6)	101.0	60.0	58	12 (2t)	6		308.0	74.0	58	226.0	60 (10t)	6	

**Table 6:** Material-property-test results.

Thickness/mm	*E*/10^5^ MPa	*F_y_*/MPa	*F_u_*/MPa	Elongation/%
3.00	1.90	296	397	34.6
3.75	1.95	294	445	36.8
6.00	2.05	292	426	30.9
8.00	2.14	288	448	32.5

**Table 7:** Shake-table-loading protocol.

Condition	Ground Motion	Earthquake Intensity	PGA/gal	
			Set Value	Measured Value
W-1	White noise	-	Very small	
Sy-1	Sy	8-degree frequent intensity	188.3	87.9
Sy-2	Sy		188.3	225.8
W-2	White noise	-	Very small	
Sy-3	Sy	8-degree basic intensity	538.0	295.4
Sy-4	Sy		538.0	671.4
W-3	White noise	-	Very small	
Sy-5	Sy	9-degree rare intensity	1668.0	1914.8
Sy-6	Sy		1668.0	1952.6
W-4	White noise	-	Very small	
Sy-7	Sy	3 continuous motions	2218.4	2252.9
Sy-8	Sy	3 continuous motions	2218.4	2494.0
Sy-9	Sy	3 continuous motions	2351.9	2748.6
Sy-10	Sy	5 continuous motions	2351.9	2516.0
W-5	White noise	-	Very small	

**Table 8:** Corresponding period of different-order vibration modes of CBF models (unit: s).

	Model	P1	S1	S2
Period				
4-story	1st	0.570	0.561	0.557
	2nd	0.202	0.197	0.194
	3rd	0.125	0.122	0.121
10-story	1st	1.630	1.592	1.568
	2nd	0.512	0.496	0.489
	3rd	0.272	0.264	0.261

## Data Availability

Some or all data, models, or code that support the findings of this study are available from the corresponding author upon reasonable request.
